# Ibrutinib and novel BTK inhibitors in clinical development

**DOI:** 10.1186/1756-8722-6-59

**Published:** 2013-08-19

**Authors:** Akintunde Akinleye, Yamei Chen, Nikhil Mukhi, Yongping Song, Delong Liu

**Affiliations:** 1 Division of Hematology/Oncology, Department of Medicine, New York Medical College, Valhalla, New York 10595, USA; 2 Department of Hematology, Xiamen Zhongshan Hospital, Xiamen University, Xiamen, China; 3 Institute of Hematology, Zhengzhou University Affiliated Tumor Hospital, Zhengzhou, China

## Abstract

Small molecule inhibitors targeting dysregulated pathways (RAS/RAF/MEK, PI3K/AKT/mTOR, JAK/STAT) have significantly improved clinical outcomes in cancer patients. Recently Bruton’s tyrosine kinase (BTK), a crucial terminal kinase enzyme in the B-cell antigen receptor (BCR) signaling pathway, has emerged as an attractive target for therapeutic intervention in human malignancies and autoimmune disorders. Ibrutinib, a novel first-in-human BTK-inhibitor, has demonstrated clinical effectiveness and tolerability in early clinical trials and has progressed into phase III trials. However, additional research is necessary to identify the optimal dosing schedule, as well as patients most likely to benefit from BTK inhibition. This review summarizes preclinical and clinical development of ibrutinib and other novel BTK inhibitors (GDC-0834, CGI-560, CGI-1746, HM-71224, CC-292, and ONO-4059, CNX-774, LFM-A13) in the treatment of B-cell malignancies and autoimmune disorders.

## Introduction

Identifying novel mediators that regulate the growth and death of cancer cells has facilitated the development of more effective anti-cancer agents that have revolutionized treatment options and clinical outcomes in cancer patients [1-4]. For instance, rituximab, a first-in-class chimeric monoclonal antibody (MoAb) targeting CD 20 molecule, has had clear impact on response rates and survival outcomes, and has become a standard component of treatment regimens for many patients with B-cell non-Hodgkin’s lymphomas (NHLs) [5-7]. MoAbs targeting CD 19 molecule are also rapidly moving through clinical trials [8]. In recent times, Bruton’s tyrosine kinase (BTK), a crucial terminal kinase enzyme in the B-cell antigen receptor (BCR) signaling pathway has emerged as a novel target [9]. This downstream signal transduction protein is a critical effector molecule that governs normal B-cell development, differentiation and functioning, and has also been implicated in initiation, survival and progression of mature B-cell lymphoproliferative disorders [10].

Ibrutinib, a novel BTK-targeting inhibitor, has shown significant activities across a variety of B-cell neoplastic disorders and autoimmune diseases in preclinical models and clinical trials [11]. However, additional research is necessary to identify the optimal dosing schedule, as well as patients most likely to benefit from BTK inhibition. This review provides a general overview of three main topics: 1) BTK signaling pathway in B-cell lymphopoiesis with emphasis on its role in the pathogenetic mechanisms that underlie B-cell lymphoproliferative disorders; 2) Novel BTK inhibitors in preclinical and clinical development. and 3) Preclinical models and clinical experiences with ibrutinib and other BTK inhibitors in the treatment of various B-cell disorders and autoimmune disorders.

### BTK signaling pathway, B-cell lymphopoiesis, and tumorigenesis

**Figure 1:**
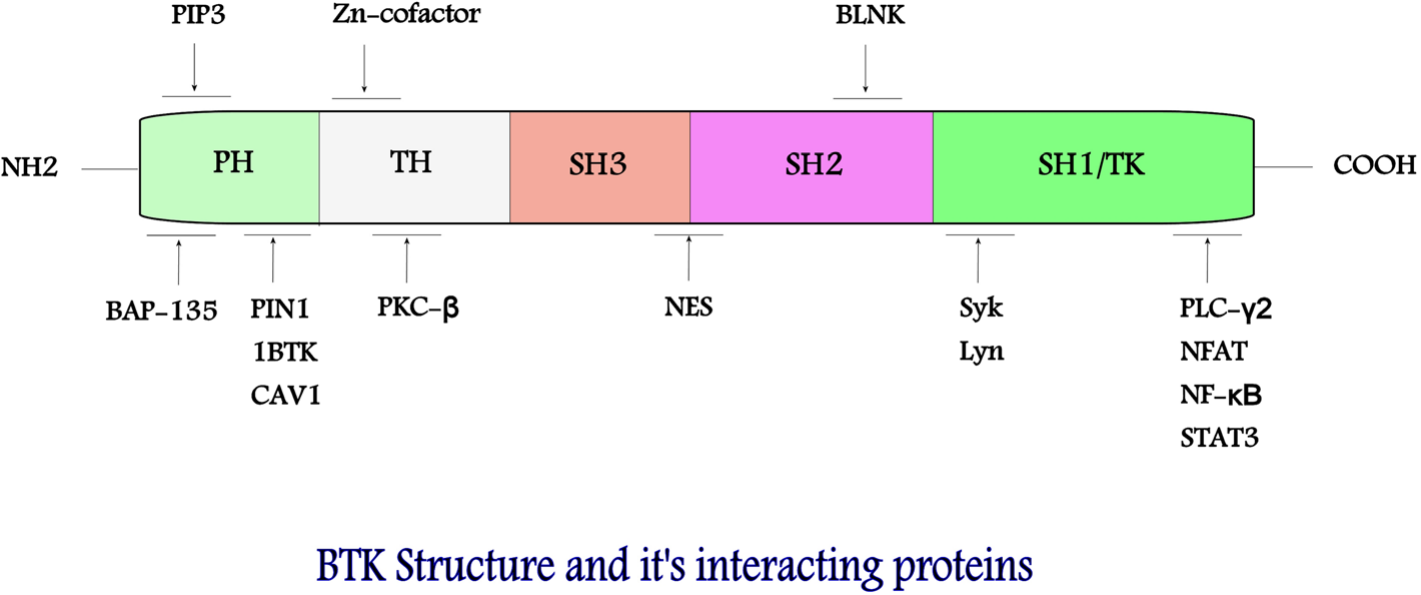
**BTK structure.** BTK belongs to the Tec family of protein tyrosine kinases and is composed of the PH (pleckstrin homology), TH (Tec homology), SH3 (Src homology 3) SH2 (Src homology 2), and SH 1/TK (Src homology1/Tyrosine kinase) domains. Binding sites for BTK substrates, inhibitors, and upstream molecules are shown in the diagram.

BTK, also known as agammaglobulinemia tyrosine kinase (ATK) or B-cell progenitor kinase (BPK), is a non-receptor tyrosine kinase that was initially identified as the defective protein in human X-linked agammaglobulinemia (XLA) [12,13]. The protein is predominantly expressed in B-lymphocytes at various stages of development (except in terminally differentiated plasma cells), and less commonly in myeloid and erythroid progenitor cells [14]. It is encoded by the *XLA* gene that maps to a 37 kb DNA fragment on chromosome Xq22 [15,16]. BTK is a member of the Tec family of protein tyrosine kinases. The Tec family has five members and is the second largest family of cytoplasmic tyrosine kinases. BTK has domains of pleckstrin homology (PH), Tec homology (TH), Src homology 3 (SH3), Src homology 2 (SH2), and tyrosine kinase or Src homology 1 (TK or SH1) (Figure 1) [17]. The PH domain contains the binding site for transcription factor BAP-135/TFII-I [18], harbors the inhibitory segment for downregulators such as PIN 1, IBTK (inhibitor of BTK) [19], and also mediates BTK’s interaction with second messenger phosphatidylinositol 3,4,5-trisphosphates (PIP_3_) [20]. Adjacent to the PH domain is a segment of 80 amino acid residues denoted as the TH domain. The TH domain houses conserved regions designated as BTK motif (zinc cofactor binding site) and proline-rich stretch [21], and serves as a major determinant binding site for protein kinase C-beta (PKC-β) [22]. Initial activation (trans-phosphorylation) of BTK takes place in the activation loop located in the SH1/TK domain; however further activation occurs within the SH3 and SH2 domains, which contains major autophosphorylation sites [23,24]. These Src homologous domains also contain the nuclear localization signals (NLS) and nuclear export sequence (NES) required for nucleocytoplasmic shuttling of BTK [25]. In addition to the activation loop, the ATP binding site, the catalytic apparatus, and the allosteric inhibitory segments are also situated in the SH1/TK domain [26].

BTK functions downstream of multiple receptors including growth factors, B-cell antigen, chemokine, and innate immune receptors, and thereby initiates a diverse range of cellular processes, such as cell proliferation, survival, differentiation, motility, angiogenesis, cytokine production, and antigen presentation [27-30]. In steady-state conditions, BTK is predominantly cytosolic, un-phosphorylated and catalytically inactive [20]. BTK activation is a complex process and a critical step in this process requires translocation of BTK to the plasma membrane [20].

**Figure 2:**
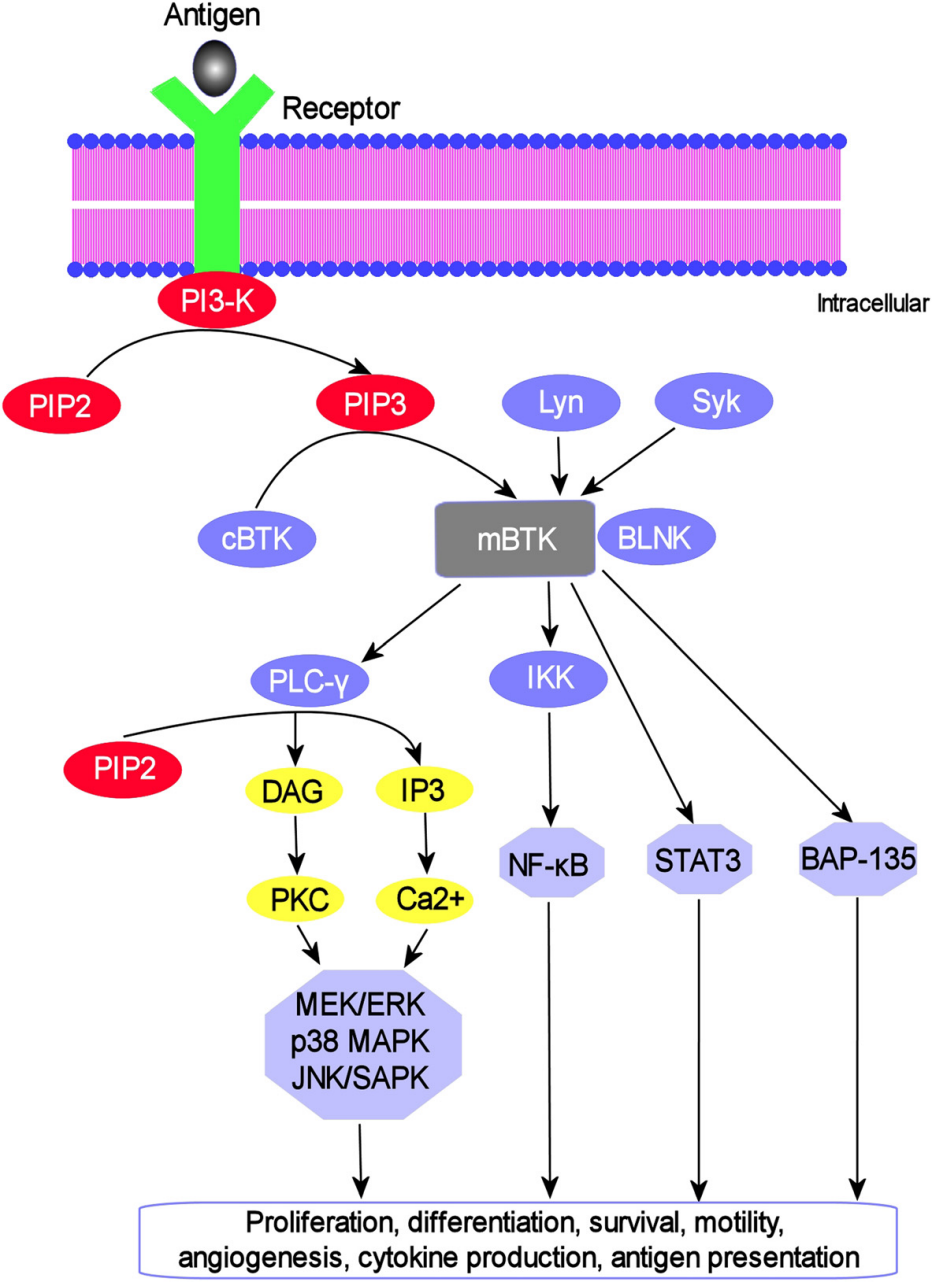
**BTK signaling pathway.** BTK translocates to the plasma membrane by interacting with PIP3 to become membrane-bound where it undergoes sequential activation through trans-phosphorylation by Lyn and Syk kinases, followed by autophosphorylation. The downstream substrates of activated BTK and their associated signaling cascades are indicated.

Upon engagement by their corresponding ligands, activated receptors recruit and phosphorylate intracellular signal transducer enzyme, phosphatidylinositol 3-kinase (PI3K), which then acts on membrane-bound phosphatidylinositol 4,5-bisphosphate (PIP2) to generate second messenger phosphatidylinositol 3,4,5-trisphosphates (PIP_3_) [20]. PIP_3_ binds to BTK’s PH domain and recruits BTK to the plasma membrane, where BTK is initially trans-phosphorylated at Tyr-551residue by Syk and Lyn kinases [31]. BTK then undergoes autophosphorylation reaction at Tyr-223 residue to become physiologically active [24]. Activated BTK can interact with adapter protein BLNK/SLP65 through its SH2 domain. The complex can then activate phospholipase C (PLC)-γ2 [32], triggering a cascade of events that culminates in sustained intracellular calcium influx and indirect activation of downstream transcriptional signaling such as MEK/ERK, p38 MAPK, and JNK/SAPK pathways (Figure 2) [27,28,33-35]. Other downstream substrates of BTK include transcription factors BAP-135/TFII-I, NFκB, ARID3A, STAT3 and NFAT, where BTK plays a critical role in direct transcription regulation and the expression of hundreds of genes [29,31,34,36]. Under certain physiological conditions, BTK can translocate to the nucleus and activate the transcription of specific target genes [25], but BTK itself does not bind directly to the DNA.

PKC-β can directly phosphorylate BTK’s TH domain at Ser-180, resulting in shuttling of BTK back to the cytoplasmic compartment [22]. Interactions with PIN1, SH3BP5, CAV1 and IBTK also lead to dramatic down-regulation of the kinase activity of BTK [19,37-39].

BTK plays indispensable roles in B-cell lymphopoiesis. It orchestrates orderly development and differentiation of immature B-cells to mature forms through activation of positive cell cycle regulators and differentiation factors [17,28], and also controls proliferation and survival of B-cells by regulating expression of pro- and anti-apoptotic proteins [40-42].

Aberrant activation of the BTK-dependent pathways has been implicated in maintaining malignant phenotype in a wide variety of malignancies. Basal growth, survival, and cancer progression in mature B-cell lymphoproliferative disorders appear to be promoted by dysregulated BTK activity [10,43,44]. Constitutive BTK activation represents an absolute prerequisite for CLL development and enhances leukemogenesis in mouse models of CLL [45]. In addition, altered BCR-BTK signaling promotes cell survival in the activated B-cell-like (ABC) subtype of DLBCL [46]. Somatic gain-of-function mutations in BTK have also been identified in colorectal carcinoma [47], acute lymphoblastic leukemia (ALL) [48], and chronic myeloid leukemia (CML) [49].

## BTK inhibitors (BTKi) in clinical trials and preclinical development

Small molecules with BTK-inhibitory property have emerged as promising therapeutic agents for the treatment of hematological malignancies and autoimmune disorders [50-52]. As such, a number of compounds, such as ibrutinib, GDC-0834, HM-71224, CC-292, and ONO-4059, have progressed through advanced preclinical development to clinical trials [http://clinicaltrials.gov].

## Ibrutinib

### Preclinical studies of ibrutinib

Ibrutinib (formerly PCI-32765) is an orally bioavailable, first-in-class, highly potent small molecule inhibitor with subnanomolar activity (IC50, 0.5 nM) against BTK (Table 1) [11]. It selectively binds to Cys-481 residue in the allosteric inhibitory segment of BTK (TK/SH1 domain), and irreversibly blocks its enzymatic activity [53]. The compound also abrogates the full activation of BTK by inhibiting its autophosphorylation at Tyr-223 [11]. In-vitro studies showed that ibrutinib induces dose- and time-dependent cytotoxicity in CLL tumor cell lines via activation of caspase-3 dependent apoptotic pathway [9]. Ibrutinib also inhibits DNA replication [54], suppresses TLR signaling-mediated proliferation [9], and blocks pro-survival pathways in CLL cells by downregulating CCL3 and CCL4 expression [54]. More so, the compound antagonizes BTK-dependent chemotaxis to CXCL12 and CXCL13 [55]. Ibrutinib interferes with proliferation and survival in B-ALL cell lines including Ph+/BCR-ABL1 positive cells [56]. In a xenografted TCL1 mouse model of CLL, ibrutinib dosed at 25 mg/kg/day delayed disease progression [54]. In-vivo studies by Honigberg et al. using MRL-Fas (lpr) lupus mouse models demonstrated that ibrutinib-induced BTK inhibition is associated with reduced autoantibody production and suppression of kidney disease development [11].

**Table 1:** BTK Inhibitors in preclinical and clinical development

**BTK inhibitor**	**Stage of development**	**Disease(s)**	**IC**_**50**_	**Reference**
Ibrutinib	Phase II/III	CLL/SLL, MCL, WM, ABC-DLBCL, MM	0.5 nM	[54,57,58,60]
GDC-0834	Phase I	Rheumatoid Arthritis	5.9 nM	[70,71]
RN-486	Preclinical	Rheumatoid Arthritis, SLE	4.0 nM	[72,73]
CGI-560	Preclinical	NR	400 nM	[74]
CGI-1746	Preclinical	Rheumatoid Arthritis	1.9 nM	[74]
HM-71224	Phase I	Rheumatoid Arthritis	NR	[75]
CC-292	Phase I	CLL/B-NHL	< 0.5 nM	[76-78]
ONO-4059	Phase I	CLL	2.2 nM	[79]
CNX-774	Preclinical	Autoimmune Diseases, B-cell NHL	< 1 nM	[80]
LFM-A13	Preclinical	B-cell NHL	17.2 μM	[81-83]

*NR* not reported.

### Clinical trials of ibrutinib

Ibrutinib is highly efficacious and safe, and has now entered phase III clinical trials for a variety of B-cell neoplasms. In an initial, multi-institutional phase I dose-escalating study, two dosing schedules of ibrutinib was tested in patients with relapsed or refractory FL, SLL/CLL, MCL, MZL, DLBCL, and WM who had failed at least one previous therapy [57]. Fifty-six patients received oral ibrutinib at doses of 1.25, 2.5, 5, 8.3, or 12.5 mg/kg daily on a 28 days on, 7 days off schedule (35-day cycle), or continuous daily dosing of 8.3 mg/kg or 560 mg until disease progression (PD) or unacceptable toxicity. After a median of 5 cycles of treatment, only two dose-limiting toxicities (DLTs) occurred and ibrutinib was found to be safe and well tolerated. The most common adverse effects were grade 1 or 2 non-hematologic toxicities, which included rash, nausea, fatigue, diarrhea, muscle spasms/myalgia and arthalgia. Hematologic toxicities were less common, and included grade 3 to 4 neutropenia (12.5%), thrombocytopenia (7.2%), and anemia (7.1%). In CLL patients, ibrutinib treatment is characteristically associated with rapid resolution of enlarged lymph nodes along with a surge in peripheral blood lymphocytosis. Of 50 patients evaluated for response, an overall response rate (ORR) of 60% was achieved across all histological types with the best efficacy demonstrated in patients with MCL (78%) and SLL/CLL (79%). The responses lasted for at least 10 months. Notably, a median progression free survival (PFS) of 13.6 months was also achieved [57]. Though both intermittent and continuous dosing schedules demonstrated similar efficacy and toxicity profiles, the study favored continuous dosing for phase II studies due to possibility of reversed biologic activity with intermittent therapy.

Given the high efficacy of ibrutinib in the precedent phase I study, a larger study was done to investigate the activity of single-agent ibrutinib in 111 heavily-pretreated patients with relapsed or refractory MCL [58]. Patients received a daily dose of 560 mg orally in a continuous 28-day cycles until disease progression or unacceptable toxicities. After a median follow-up of 15.3 months, the ORR was 68% (21% CR, 47% PR) with median response duration of 17.5 months, and was independent of patients’ baseline characteristics or risk factors. Though the median OS was not reached, the median PFS was 13.9 months. Based on these collective efficacy data, ibrutinib was granted a ‘breakthrough therapy’ designation by the FDA in February 2013 for patients with relapsed/refractory MCL, and a phase III registration trial (RAY) of ibrutinib monotherapy versus temsirolimus has been initiated in the same patient population.

Long-term tolerability and sustained antitumor activity of ibrutinib in heavily pretreated patients with refractory/relapsed FL have also been reported. Kunkel and colleagues showed that ibrutinib was well-tolerated and active, with an ORR was 55% (3 CR) and median PFS was 13.4 months [59]. Most common treatment-related side effects were dry mouth, constipation and diarrhea.

Encouraging results from the phase I study in CLL patients prompted a phase Ib/II trial, where patients with relapsed/refractory CLL/SLL (n=85), predominantly with high-risk disease, received oral ibrutinib at either 420 mg or 840 mg daily until disease progression or unacceptable toxicity [60]. Tolerability profile was acceptable as most adverse events were grade 1 or 2 diarrhea, fatigue, and URI that resolved spontaneously. Higher drug discontinuation rate occurred in the 840 mg cohort compared with the 420 mg cohort (12% versus 4%). The ORR was 71% in both cohorts, and not dependent on clinical and genomic risk factors (such as 17p deletion) present before treatment. The characteristic drug-induced re-distribution lymphocytosis that accompanies lymph node reduction was observed generally by day 7, peaked at median of 4 weeks of treatment, and then slowly declined. The responses were durable with the estimated 26-month PFS and OS rates being 75% and 83% respectively. Of note, this study initially included thirty-one treatment-naïve CLL patients older than 65 years, where ibrutinib was investigated as an upfront therapy. After a median follow-up of 16.6 months, the overall response rate was 71% (CR 10%) suggesting that ibrutinib could be a reasonable choice for newly diagnosed elderly patients with CLL [61,62].

Patients’ enrollment is underway for two phase III registration trials of single-agent ibrutinib in CLL/SLL patients to further demonstrate its impact on clinical outcomes. In RESONATE 1, ibrutinib is being compared with ofatumumab in patients with relapsed/refractory CLL/SLL, whereas RESONATE 2 is to investigate ibrutinib as frontline therapy for newly diagnosed elderly patients with CLL/SLL in comparison with chlorambucil. Interim analyses from these trials are expected during the 1st quarter 2014.

Updated interim results from an ongoing open label phase II trial showed that ibrutinib is active in patients with activated B cell-like (ABC) subtype of DLBCL harboring both CD79B and MYD88 L265P mutations [63]. Heavily pretreated patients (n=70) with relapsed/refractory DLBCL received oral ibrutinib 560 mg daily until disease progression or onset of unacceptable toxicities. The ORR was significantly greater in patients with the ABC subtype compared to those with the GCB subtype (41% versus 5%, p=0.007), suggesting preferential antitumor activity in ABC DLBCL. The median OS was 9.7 months for the ABC subtype, compared to 3.35 months for the GCB subtype. Similarly, ibrutinib has potential antitumor activities in multiple myeloma (MM), targeting both tumor cells and their supporting microenvironment. Vij and colleagues reported early reductions in plasma levels of growth factors (e.g. VEGF, EGF, and FGF), cytokines (e.g. TNFα), chemokines (CCL3, CCL4, Groα), and markers of bone turnover (e.g. sclerostin, RANKL) in patients with relapsed/refractory MM treated with ibrutinib 420 mg once daily [64].

### Clinical trials of ibrutinib in combination regimens

The promising results of single agent ibrutinib have led investigators to explore its synergistic efficacy in combination with established chemoimmunotherapy regimens with the goal of enhancing and achieving durable responses. Treatment with ibrutinib plus ACY1215, a selective histone deacetylase 6 (HDAC6) inhibitor, produced a direct synergistic antitumor effect in MCL tumor cell lines accompanied by a 3-fold increase in induction of apoptosis [65]. One of the first clinical studies addressing this purpose was a phase II study that showed ibrutinib plus rituximab (IR) was profoundly effective (ORR=85%), and shortened the duration of re-distribution lymphocytosis in CLL patients with high-risk features [66]. Adding ibrutinib to BR (bendamustine and rituximab) appear to produce a better clinical response (ORR=93%) than IR in relapsed/refractory CLL patients [67]. In a recent report of another ongoing phase Ib/II study, administration of ibrutinib in combination with ofatumumab demonstrated potent anti-leukemic activity and tolerable toxicity profile in heavily-pretreated patients with relapsed/refractory CLL/SLL [68]. Blum et al. also demonstrated excellent response rates in MCL patients treated with a combination of ibrutinib plus rituximab and bendamustine [69]. In light of these encouraging results, two phase III trials are currently accruing participants to investigate the combination of ibrutinib plus bendamustine and rituximab versus placebo plus bendamustine and rituximab in subjects with newly diagnosed MCL (SHINE), as well as in patients with refractory/relapsed CLL (HELIOS). Additional preclinical experiments and clinical trials are currently underway to further explore this strategy in other B-cell disorders [NCT01829568, NCT01569750, NCT01479842].

Given the current data, ibrutinib appears to be one of the most active single agents for CLL/SLL and MCL.

## GDC-0834

GDC-0834 is a potent, highly selective, reversible BTK inhibitor with nanomolar activity in enzyme kinetics studies. A carboxamide derivative, GDC-0834 is being developed as a potential therapeutic agent for rheumatoid arthritis (RA) [70]. The compound demonstrates effective activity against BCR- and CD40-dependent B-cell proliferation and activation, and potently inhibits immune complex-mediated inflammatory cytokine elaboration in monocytes. In collagen-induced arthritis (CIA) rat models, treatment with oral GDC-0834 dosed at 30-100 mg/kg demonstrated robust anti-arthritis effect characterized by significant dose-dependent reduction in ankle swelling, and accompanied by potent inhibition of autophosphorylation of BTK [70]. Pharmacokinetics (PK) results of a recent phase I study of GDC-0834 in healthy volunteers showed that the drug is heavily metabolized by the liver to an inactive metabolite via amide hydrolysis [71]. GDC-0834 is undergoing further clinical development to assess its safety and tolerability in patients with inflammatory arthritis.

## RN-486

A potent and competitive small molecule with reversible BTK-inhibitory property, RN-486 demonstrates subnanomolar and highly specific activity against purified BTK in enzymatic assays. It blocks BCR-mediated CD69 expression in B-cells in a dose-dependent manner. Preclinical studies also showed that RN-486 efficiently inhibits FcR-mediated TNF-α production in monocytes, and abrogates FcϵR-mediated mast cell degranulation [72]. The anti-rheumatic potential of RN-486 has been investigated in preclinical studies. In two murine models of RA, the compound demonstrates potent anti-inflammatory and disease-modifying effects characterized by reduction in pannus formation, cartilage damage and bone resorption [72]. It also abrogates type I and type III hypersensitivity responses in rats. RN-486 suppresses IgG anti-dsDNA secretion, blocks CD69 expression in response to BCR crosslinking, and completely inhibits progression of glomerular nephritis in systemic lupus erythematosus (SLE) prone NZB/W mouse models [73].

## CGI-560

CGI-560, a benzamide derivative, is a highly selective (>10 fold) but modestly potent small molecule inhibitor of BTK with an IC50 of 400 nM in enzymology assays [74]. Optimization of CGI-560 property by medicinal chemistry led to the discovery of another benzamide analogue (CGI-1746) with exquisite potency and unique BTK-inhibitory activity [74].

## CGI-1746

An exquisitely selective and ATP-competitive small molecule inhibitor with unique BTK-inhibitory property, CGI-1746 potently inhibits both auto- and trans-phosphorylation of BTK. It binds and occupies an SH3 binding pocket within the un-phosphorylated BTK and stabilizes it in this inactive enzyme conformation state [74]. In cellular assays, the compound blocks BCR-mediated B-cell proliferation and suppresses FcγRIII-induced TNFα, IL-1β and IL-6 production in macrophages. CGI-1746 demonstrated robust anti-arthritic activity in experimental mouse models evident by diminished cytokine and autoantibody levels in the joints [74].

## HM-71224

HM-71224 is a novel, oral, small molecule BTK inhibitor that is being developed by Hanmi pharmaceuticals [75]. The compound has progressed into phase I clinical testing, and its PD, PK, safety, and tolerability are being assessed in healthy volunteers in Korea and the Netherlands [NCT01765478].

## CC-292

CC-292 (formerly AVL-292) is an orally bioavailable acrylamide derivative with potent, irreversible anti-BTK activity (IC50 <0.5 nM) in biochemical kinase assays. The small molecule inhibitor abolishes BCR signaling in Ramos human Burkitt's lymphoma cell line by covalently binding to BTK, and selectively inhibits its autophosphorylation as well as activation of PLCγ2 and other downstream substrates of BTK [76]. When dosed orally five times a week for 6 weeks, CC-292 decreased tumor burden in preclinical xenografts MM mouse models of human Luc-GFP^+^-MM.1S myeloma cell line [77]. In established CIA mouse models, treatment with CC-292 dosed at 3, 10 and 30 mg/kg produced dose-dependent resolution of clinical signs and histopathologic features of inflammatory joint disease including reduction in joint swellings and redness, and regression of pannus formation [76]. Clinical progress has been seen with CC-292. In normal healthy volunteers who received 2 mg/kg CC-292, PK analysis showed that the compound was rapidly absorbed, achieving peak plasma concentrations within 30–120 minutes, and demonstrating a median terminal elimination half-life of 1.9 hours. Analysis of BTK activity in same study subjects indicated that the drug remained active for a prolonged duration after its plasma levels had declined to undetectable levels [76]. Given these data, CC-292 was advanced to phase Ib clinical testing in patients with B-cell disorders. Updated results from a dose-finding study of CC-292 in patients (n=86) with relapsed or refractory CLL and B-NHL revealed that the agent was generally well-tolerated at doses ranging from 125-1000 mg daily, and at 375 and 500 mg twice daily [78]. The most common treatment-emergent adverse effects were diarrhea, fatigue, headaches and muscle spasms. Three DLTs including thrombocytopenia, pneumonitis, and altered mental status were reported. Of 50 CLL patients evaluated for response, seventeen (34%) achieved PR. Multiple ongoing phase I studies are expected to provide additional safety results on CC-292 as a single agent or combination with other agents in patients with a wide variety of B-cell lymphoproliferative disorders [NCT01766583, NCT01744626, NCT01732861].

## ONO-4059

ONO-4059 is a highly selective, orally bioavailable inhibitor of BTK kinase activity with a potency (IC50) of 2.2 nM. The compound covalently binds to BTK, and reversibly blocks BCR signaling and B-cell proliferation and activation. Like CC-262, ONO-4059 demonstrated therapeutic efficacy in a mouse CIA model by suppressing generation of inflammatory chemokines and cytokines including IL-6, IL-8, and TNFα by monocytes, and accompanied by regression of cartilage erosion, bone damage, and pannus formation [79]. The data indicate that ONO-4059 may have a potential benefit for the treatment of patients with RA. In view of its anti-proliferative activity in B-cells, ONO-4059 has progressed into phase I clinical trials in CLL/NHL patients with relapsed/refractory disease.

## CNX-774

CNX-774 is another orally available, small molecule inhibitor with irreversible BTK-inhibitory property. CNX-774 is highly selective for BTK, and forms a ligand-directed covalent bond with the Cys-481 residue within the ATP binding site of the enzyme. In biochemical and cellular assays, CNX-774 demonstrates potent inhibitory activity towards BTK with an IC50 of <1nM and 1-10nM respectively [80]. The compound has progressed to advanced preclinical development and additional in-vitro and in-vivo data are awaited.

## LFM-A13

LFM-A13 is a novel, first-in-class, dual BTK/Polo-like kinases (PLK) inhibitor with anti-proliferative, pro-apoptotic, and chemosensitising effects in leukemia/lymphoma and breast cancer cells [4,81,82]. A leflunomide metabolite analogue, LFM-A13 binds favorably to the catalytic site within the kinase domain of BTK, and exhibits an inhibitory potency (IC50) of 17.2microM in cell-free kinase assays [83]. The compound is highly selective for BTK, and specifically inhibits cellular BTK activity in chicken lymphoma B18.2 B-cells and human *NALM*-*6* leukemic pre-B cells in a dose-dependent fashion [83]. In human Ph+ ALL-1 and NALM-6 pre-B ALL cell line, treatment with LFM-A13 enhances the sensitivity of the cells to both ceramide- and vincristine-induced apoptosis [83]. Dosed at levels ranging from 10 to 80 mg/kg, LFM-A13 was not toxic to xenografted murine leukemia model [81]. The compound down-regulates BTK signaling in myeloma cells evident by reduced in vivo homing of myeloma cells to bone, prevention of myeloma-induced bone resorption, and moderate suppression of myeloma growth in primary myeloma-bearing SCID-rab mice [84]. These preclinical data provide the rationale for future clinical development of LFM-A13 as a new therapeutic agent for B-cell lymphoproliferative disorders.

## Conclusion and future directions

Ibrutinib, a novel BTK-targeting inhibitor, has shown significant activities across a variety of B-cell neoplastic disorders and autoimmune diseases in preclinical models and clinical trials. The data from clinical trials on CLL and mantle cell lymphoma are particularly encouraging. Novel BTK inhibitors, GDC-0834, HM-71224, CC-292, and ONO-4059, CNX-774, LFM-A13, are under active preclinical and clinical investigation. Among these, LFM-A13 represents a first-in-class dual BTK-PLK inhibitor. These novel inhibitors will provide new targeted therapy not only for B-cell lymphomas, but also for autoimmune disorders. Further research into combination of novel small molecule inhibitors against different signaling pathways as well as combination of these inhibitors with other biological and biochemical compounds will likely enhance their clinical efficacy [34,85].

## Competing interest

DL is a clinical investigator participating in SHINE and HELIO trials sponsored by Janssen. The remaining authors have no conflicts of interest.

## Authors’ contributions

DL and AA were responsible for study design, data collection and drafting the manuscript. NM designed the figures. All authors have participated in manuscript development, revisions and approved the final manuscript.
